# “Employment and arthritis: making it work” a randomized controlled trial evaluating an online program to help people with inflammatory arthritis maintain employment (study protocol)

**DOI:** 10.1186/1472-6947-14-59

**Published:** 2014-07-21

**Authors:** Erin C Carruthers, Pamela Rogers, Catherine L Backman, Charles H Goldsmith, Monique A Gignac, Carlo Marra, Judy Village, Linda C Li, John M Esdaile, Diane Lacaille

**Affiliations:** 1 Arthritis Research Centre of Canada, 5591 No. 3 Rd, Richmond, BC V6X 2C7, Canada; 2 Department of Occupational Science and Occupational Therapy, University of British Columbia, T325-2211 Wesbrook Mall, Vancouver, BC V6T 2B5, Canada; 3 Faculty of Health Sciences, Simon Fraser University, 8888 University Drive, Burnaby, BC V5A 1S6, Canada; 4 Dalla Lana School of Public Health, University of Toronto, 10 MP-328, 399 Bathurst St, Toronto, ON M5T 2S8, Canada; 5 School of Pharmacy, Memorial University, Health Sciences Centre, 300 Prince Philip Drive, St. John’s, NL A1B 3 V6, Canada; 6 School of Population and Public Health, University of British Columbia, 2206 East Mall, Vancouver, BC V6T 1Z3, Canada; 7 Department of Physical Therapy, University of British Columbia, 5591 No. 3 Rd, Richmond, BC V6X 2C7, Canada; 8 Division of Rheumatology, University of British Columbia, 5591 No. 3 Rd, Richmond, BC V6X 2C7, Canada

**Keywords:** Inflammatory arthritis, Employment, Worker productivity, Work disability, eLearning program, Self-management, Ergonomics, Vocational counselling, Health education, Health promotion

## Abstract

**Background:**

Arthritis and musculoskeletal conditions are the leading cause of long-term work disability (WD), an outcome with a major impact on quality of life and a high cost to society. The importance of decreased at-work productivity has also recently been recognized. Despite the importance of these problems, few interventions have been developed to reduce the impact of arthritis on employment. We have developed a novel intervention called “Making It Work”, a program to help people with inflammatory arthritis (IA) deal with employment issues, prevent WD and improve at-work productivity. After favorable results in a proof-of-concept study, we converted the program to a web-based format for broader dissemination and improved accessibility. The objectives of this study are: 1) to evaluate in a randomized controlled trial (RCT) the effectiveness of the program at preventing work cessation and improving at-work productivity; 2) to perform a cost-utility analysis of the intervention.

**Methods/Design:**

526 participants with IA will be recruited from British Columbia, Alberta, and Ontario in Canada. The intervention consists of a) 5 online group sessions; b) 5 web-based e-learning modules; c) consultations with an occupational therapist for an ergonomic work assessment and a vocational rehabilitation counselor. Questionnaires will be administered online at baseline and every 6 months to collect information about demographics, disease measures, costs, work-related risk factors for WD, quality of life, and work outcomes. Primary outcomes include at-work productivity and time to work cessation of > 6 months for any reason. Secondary outcomes include temporary work cessation, number of days missed from work per year, reduction in hours worked per week, quality adjusted life year for the cost utility analysis, and changes from baseline in employment risk factors. Analysis of Variance will evaluate the intervention’s effect on at-work productivity, and multivariable Cox regression models will estimate the risk of work cessation associated with the intervention after controlling for risk factors for WD and other important predictors imbalanced at baseline.

**Discussion:**

This program fills an important gap in arthritis health services and addresses an important and costly problem. Knowledge gained from the RCT will be useful to health care professionals, policy planners and arthritis stakeholders.

**Trial registration:**

ClinicalTrials.gov NCT01852851; registered April 13, 2012; first participant randomized on July 6, 2013.

## Background

Work disability (WD) is a common and early outcome of inflammatory arthritis (IA), with a major impact on quality of life and a high cost to those affected, their families and society [1,2]. Arthritis and musculoskeletal conditions are the leading cause of long-term WD in many countries, including Canada and the US [3,4]. Recent studies have also drawn attention to the importance of decreased productivity while at work, or presenteeism [5]. Despite the importance of the problem, there has been little research on interventions to reduce the impact of arthritis on employment [6-9]. Lack of employment services has also been identified by people living with arthritis as an important unmet need in qualitative studies [10-12]. It represents an important gap from a clinical care and a health services research perspective.

To close this care gap, we have developed and pilot tested a novel intervention targeting employed people with IA, to prevent WD and improve at-work productivity [13]. Our “Employment and Arthritis: Making It Work Program” is unique in that it combines the benefits of group sessions and self-learning modules aimed at enhancing self-management of work-related problems, and individualized assessments by employment-related health professionals, including an ergonomic assessment by an occupational therapist (OT) and job retention vocational rehabilitation counselling (VRC). Program development was based on a strong theoretical background, the self-management approach proven effective in arthritis management [14,15] and the precede-proceed model [16-18], a validated model successfully used for developing health education programs. Program content was selected to modify known risk factors for WD and problems at work identified in initial focus groups [10]. A proof of concept study of the intervention showed favourable results after 12 months of follow up [13]. The program has now been converted to a web-based format to allow broader dissemination of the program, and to address feedback from participants who found it difficult to attend sessions at the end of a work day, given the fatigue and difficulty commuting associated with their IA. This evolution is consistent with recent trends in adult learning, health education and in self-management programs [19-31].

The objectives of this study are: 1) to evaluate, in a randomized controlled trial (RCT), the effectiveness of the program at preventing work cessation and improving at-work productivity compared to a control group receiving usual care; 2) to perform a cost-utility analysis of the intervention.

## Methods/design

### Study design

Our study design is consistent with CONSORT recommendations for RCTs, 2010 update [32] and extension for trials of non-pharmacological treatments [33]. Participants will be stratified by location and arthritis type and randomized 1:1 to receive either our program, or “usual care” as initiated by their physician and supplemented by printed material about work and arthritis. Participants will be followed for 5 years, the estimated time required, according to our power calculations, to observe a difference in work cessation similar to that in Allaire’s RCT [34]. After 2 years, analysis of our first primary outcome, at-work productivity, will be performed, along with an interim analysis of the second primary outcome, work cessation. If a significant difference in work cessation is observed between the two groups, the RCT will be terminated prematurely. Because of the nature of the intervention, participants cannot be blinded. Ethics approval was obtained from the University of British Columbia Research Ethics Board (H11-03527).

### Study sample

526 participants will be recruited from British Columbia, Alberta, and Ontario. To be eligible to participate, individuals must: 1) have a physician confirmed diagnosis of IA (including rheumatoid arthritis, psoriatic arthritis, systemic lupus erythematosus, or spondyloarthropathies); 2) Be between the ages of 18 and 59 years; 3) Be able to read and write in English without the assistance of a translator; 4) Be currently employed (full time, part time, self-employed, or contract work); 5) Be at risk of job loss, defined as answering yes to: “Do you have any concern about your arthritis affecting your ability to work now, or in the next five years?”; 6) Have access to a personal computer with high-speed, wired-in internet connection and have access to, or willingness to purchase, a webcam and headset for the virtual group meetings and VRC assessments; 7) Be willing to travel for one visit with an OT.

### Randomization

Randomization will be stratified by study centre (3 levels) and type of arthritis (4 levels). In each stratum, we will use blocked randomization, with random and variable block sizes, to ensure optimal balance between trial arms, while avoiding the risk of study personnel ‘guessing’ the next allocation. Randomization will be performed centrally, in Vancouver; by the biostatistician (CG) using a customized program in the R programming language, after the study coordinator has confirmed eligibility and received informed consent. To ensure concealment of the randomization list from those making eligibility decisions, allocations will be available for one patient at a time.

### Intervention

The intervention consists of three components:

1) Five interactive web-based eLearning modules, completed individually by participants in between the biweekly group sessions. The modules cover relevant topics such as work and arthritis, management of IA, dealing with fatigue and stress at work, effective communications skills, disclosing one’s arthritis diagnosis, optimizing interpersonal relationships at work, job accommodations, and vocational counselling. In addition to providing didactic information, reflective exercises improve self-awareness and allow participants to apply the concepts learned to their own personal situation. Self-management techniques and other relevant skills are also taught and practiced.

2) Five 2-hour biweekly group sessions conducted as virtual real-time group meetings, with 10–12 participants per group, led by a facilitator with knowledge of arthritis and experience leading self-management or education programs. The group interactions allow participants to share experiences, learn from each other, practice skills, and report back to the group on the progress of their action plans. These interactions were highly valued by participants of our pilot test, who described experiencing validation by hearing from others living similar situations, benefiting from others’ experiences and solutions, receiving peer support, and being motivated to implement changes by having to report back to the group [13].

3) Consultations with an OT and a VRC. After completing the web-based modules and group meetings, participants have an in-person consultation with an OT for an ergonomic assessment of their work. The OT uses a standardized tool we developed for people with IA called the Ergonomic Assessment Tool for Arthritis (EATA) [35], to identify ergonomic problems and risks, and recommend ergonomic modifications as job accommodations. A telephone follow-up is performed one month later to discuss implementation of recommendations and help overcome barriers. A vocational counsellor provides “job retention” VRC, using a standardized guide developed for our program, with an initial consultation performed using web technology and a follow-up one month later by telephone. The assessment focuses on identifying problems at work and developing solutions, evaluating the need for job accommodations and helping in the process of requesting and obtaining job accommodations, short and long-term planning such as determining if additional training would facilitate long-term goals, as well as discussing individual issues such as the decision to disclose one’s arthritis, or dealing with interpersonal difficulties with co-workers or supervisors.

### Control group

The control group will receive printed materials providing information about arthritis and employment available from arthritis websites and non-profit organizations. They will also receive “usual care” from their physicians and allied health professionals. Data will be collected, in follow-up assessments, on information and support received to address employment issues. To minimize loss to follow-up, all controls will be offered the intervention at study end.

### Data collection

Data will be collected on demographics, disease variables, co-morbidities, quality of life, costs, work-related risk factors for WD, and work outcomes, using a self-administered online questionnaire. Work outcomes, costs and quality of life will be assessed every 6 months, and other data once yearly. To evaluate co-interventions received in both groups, data will also be collected every 6 months about IA treatment received, “usual care” received from physicians and allied health professionals, information sought about employment issues, use of services specifically targeted at employment, and job accommodations made.

### Outcome measures

#### ***Primary work outcomes***

The first primary outcome will be presenteeism, or at-work productivity, measured over 2 years, using the Work Instability Scale (WIS-RA) [36]. Developed for RA, the WIS measures mismatch between functional ability and work demands, and was designed to identify individuals at high risk of work loss warranting referral for vocational assessment. It has been shown to have good psychometric properties, including construct validity [37], and be effective at predicting future work transitions [38]. However, a drawback of the WIS is that it does not permit the calculation of associated costs. Therefore, for the purpose of the cost-utility analysis, at-work productivity will be measured using the Work Productivity and Activity Impairment – Specific Health Problem (WPAI-SHP) scale [39]. The WPAI-SHP measures overall impairment in activities (from work time missed, impairment while working, and activity impairment outside of work) due to a health problem, expressed as percent impairment, which can be used to calculate associated cost. Statistically significant improvement in both the WIS and the WPAI-SHP were observed in our pilot study.

The second primary outcome will be time to work cessation, measured over 5 years of follow up. Work cessation is defined as complete cessation of work for more than 6 months, the standard duration used by insurance companies to define permanent WD, and also consistent with definitions of WD in the employment literature [40]. This definition will capture WD from all health reasons (i.e. not only related to arthritis), including early retirement, work cessation for personal reasons, and prolonged unemployment periods when people are looking for another job, but will not capture temporary WD, sick leave, or short periods of unemployment of less than 6 months duration. Whether or not work cessation should be restricted to arthritis related work cessation is an issue of debate in the arthritis employment literature. Because of difficulties attributing work cessation to a single cause (i.e. the decision is often multifactorial and arthritis may have played some role in the decision), it is generally recommended that all-cause work cessation be measured in studies including a control group [41].

#### ***Secondary work outcomes***

Secondary work outcomes will include: 1) temporary work cessation for any reason (i.e. sick leave, temporary WD, and short periods of unemployment lasting more than 2 months but less than 6 months); 2) occasional work absence (i.e. number of days missed from work per year); 3) reduction in usual amount of time worked (i.e. reduction in hours per week worked); 4) At-work productivity measured using a new scale currently being developed and validated based on a combination of the Workplace Activity Limitations Scale (WALS) and Work Limitations Questionnaire (WLQ); 5) Quality adjusted life year (QALY), will be measured using EQ-5D and SF-6D, 2 years post intervention, for the cost-utility analysis; 6) Changes from baseline in job satisfaction, self-efficacy at work, work-related risk factors for WD (job physical demand, job autonomy, difficulty commuting, self-employment and support from co-workers, employers and family), job accommodations, and job type will also be evaluated, as exploratory analyses, in an attempt to understand the effect of the intervention on mediators of work disability.

### Sample size and analysis

Sample size was calculated for the primary outcome requiring the largest sample, i.e. time to work cessation. It was performed with PASS software [42] for time-to-event analyses. We assumed 5 years of follow-up and a rate of work cessation of 12.77% per year in the control group, based on the 40% work loss observed in controls over 4 years in Allaire’s RCT of VRC [34], and assuming an exponential distribution of time to work loss. A sample size of 420 subjects (210 per group) completing the study is necessary for 80% power, a 2-tailed Cox model-based score test, equivalent to the logrank test, at alpha = 0.05, to detect a 35% relative risk reduction (HR = 0.65) in the intervention vs. control group, which was considered a minimally clinically important difference (MCID), and is moderately conservative compared to the 42% risk reduction observed in Allaire’s study (HR = 0.58, 95% CI:0.34-0.99) [34]. Assuming 20% loss to follow-up, we will recruit a total of 526 subjects (263 per group). This sample will ensure adequate power for our other primary outcome, at-work productivity.

Appropriate descriptive statistics will be used to measure baseline characteristics of the two groups. Analysis of treatment effectiveness will rely on intention-to-treat (ITT) approach, so that all patients initially randomized will be included in the analysis, regardless of their compliance or drop-outs.

For the analysis of our first primary outcome, at-work productivity measured as a continuous variable will be tested for normality using the Shapiro-Wilk test [43]. If normality assumption is rejected, an appropriate transformation (e.g. such as logarithmic) will be employed to obtain an approximately normal distribution. Next, an Analysis of Variance (ANOVA) will evaluate the effect of the intervention on WIS measures at 2 years, adjusting for the 12 strata and blocking. We will also consider an Analysis of Covariance model after inspection of the baseline values in both treatment arms for factors such as WIS score, duration of disease, physician providing RA care, age, gender and risk factors for WD. If differences at baseline are observed, we will consider them as covariates. Since the power of these analyses will likely be less than the original ANOVA, post hoc power analyses will be conducted if these appear to be clinically meaningful adjustments.

In the analysis of our second primary outcome, time-to-event (survival) methodology will compare the risk of work cessation in the intervention and control groups. Subjects employed until end of their follow-up (study end, death or time of loss to follow-up, whichever occurs earlier) will be censored. First, Kaplan Meier ‘survival curves’ and logrank test will estimate and compare the proportions of subjects who continue being employed in the intervention and control groups. Next, multivariable Cox Proportional Hazards (PH) regression model [44] will be employed to estimate the adjusted Hazard Ratio (HR) with 95% CI, associated with the intervention after controlling for potential risk factors for WD that may be imbalanced at baseline, despite randomization, and for other important predictors, such as age, gender, RA duration at baseline, and physician providing RA care, whose omission could induce bias toward the null in time-to-event analyses [45]. We will use a flexible extension of the Cox model [46], which will allow us to estimate how the HR reduction due to intervention changes over time, if the proportional hazard assumption (that the intervention effect remains constant over time) is rejected.

For analyses of secondary outcomes, time-to-event methods will be employed for dichotomous outcome variables (e.g. time to temporary work cessation), and comparison of means between intervention and control groups will be employed for continuous variables (e.g. measures of absenteeism, job satisfaction, self-efficacy and risk factors for WD), as described above for the primary outcomes.

For the second primary outcome (time to work cessation), an interim analysis will be performed at 2 years of follow-up. We will use the same method as for the primary analysis and will employ the PETO criteria to adjust the overall type I error for multiple testing [47]. Specifically, we will use a conservative type I error of 0.001 for the interim analysis, which will allow us to maximize the power of the final analyses of the 5 year outcomes, which will employ the conventional 0.05 significance level.

We will also examine the incremental costs and benefits of receiving the intervention versus standard of care. Both a cost-effectiveness analysis and a cost-utility analysis will be performed. The outcome of our analysis will be the incremental cost-effectiveness ratio (ICER), which is the difference between the mean costs of providing competing interventions divided by the difference in effectiveness. Based on the primary outcome of the RCT, we will determine the incremental cost of the intervention per work cessation avoided relative to standard treatment. Because cost-effectiveness analyses are not robust to quantity or quality of life, we will also conduct a cost-utility analysis, where the primary outcome is the quality adjusted life year (QALY). Using health utilities measured by the EQ-5D and SF-6D, we will conduct a cost-utility analysis in which the incremental cost of the intervention relative to standard therapy per QALY gained will be determined. Important aspects of economic evaluations conducted alongside an RCT are excluding protocol driven costs, and dealing with missing data due to attrition. We will follow recommendations by the International Society for Pharmacoeconomics and Outcomes Research for missing cost and effectiveness data. We will use a combination of imputation and bootstrapping to quantify uncertainty due to missing values and finite study sample size. We will use propensity scores, stratified by treatment group, for imputing missing costs and effectiveness data due to attrition.

### Limitations

Limitations are those inherent to the study design and intervention. Participating in research, especially completing questionnaires may trigger awareness of problems, leading to more active coping behaviour than “usual” in the control group. The un-blinded nature of our intervention increases the risk of contamination (administration of part of the intervention to the control group, especially referral to OT and VRC) and unequal co-intervention (i.e. unequal administration of additional measures to deal with employment problems). However, these issues would reduce our ability to observe a difference between intervention and control group and therefore would constitute a conservative bias. Also, our recruitment strategy (i.e. avoiding prospective enrolment of consecutive patients as they are seen by rheumatologists) was specifically designed to limit these risks. We will collect data to document the extent to which contamination or co-intervention occurs.

Our intervention is a complex one involving several components. Our study was designed to evaluate the effectiveness of the intervention as a whole and not to evaluate the relative effectiveness of each component. Five years of follow-up is long and increases the risk of loss to follow-up. However, this is the time required to evaluate work cessation. The importance of this outcome, in terms of quality of life and economic impact, at the personal and societal level, warrants the difficulty of a lengthy trial. Preventing work disability is the outcome most relevant to health policy planners, employers, insurance companies, and will influence the decision to fund the program as part of the multidisciplinary services offered to people with arthritis. We have designed strategies to enhance retention.

## Discussion

We have created a novel program for people with IA to improve an aspect of disease management too often ignored by health professionals, the management of employment issues. Our program is unique in that it combines the benefit of group sessions focused on improving self-management, with assessments by health professionals specifically addressing employment. The web-based technology for delivering our program is at the leading edge of trends in the field of adult education and self-management programs, and offers a number of advantages for facilitating both learning and program dissemination.

Our research initiative is one of few worldwide to develop and test the effectiveness of interventions specifically targeted at employment. Before our program can be implemented as part of routine care, it is important that its effectiveness be rigorously evaluated. The knowledge gained from this RCT will be useful for health care professionals to refer to the program, for health care policy planners to agree to fund it as part of multidisciplinary arthritis services, and for arthritis stakeholders to endorse the program, promote it and help disseminate it widely. By preventing WD, our program has the potential for great cost savings to society and huge personal impact on people’s lives.

## Competing interests

The authors declare that they have no competing interests.

## Authors’ contribution

DL, as the principal investigator, conceived the study design and developed the study protocol. All co-investigators (CB, CHG, MG, CM, JV, LCL, JME) contributed to the study design and protocol. EC contributed to implementation of the RCT and manuscript writing. PR contributed to study design and implementation of the RCT. All authors read and approved the final manuscript.

## Pre-publication history

The pre-publication history for this paper can be accessed here:

http://www.biomedcentral.com/1472-6947/14/59/prepub
